# Interaction of Functional Brain Networks Is Associated With *k*‐Clique Percolation in the Human Structural Connectome

**DOI:** 10.1002/hbm.70343

**Published:** 2025-10-23

**Authors:** Vasilii Tiselko, Olesia Dogonasheva, Artem Myshkin, Denis Zakharov, Olga Valba

**Affiliations:** ^1^ Laboratory of Complex Networks Center for Neurophysics and Neuromorphic Technologies Moscow Russia; ^2^ Centre for Cognition and Decision Making, Institute for Cognitive Neuroscience National Research University HSE Moscow Russia; ^3^ Phystech School of Applied Mathematics and Computer Science Moscow Institute of Physics and Technology Dolgoprudny Moscow Russia; ^4^ Université Paris Cité, Institut Pasteur, AP‐HP, Inserm, Fondation Pour l'Audition, Institut de l'Audition, IHU reConnect Paris France; ^5^ National Research University HSE Moscow Russia

## Abstract

The human structural connectome has a complex internal community organization, characterized by a high degree of overlap and related to functional and cognitive phenomena. We explored connectivity properties in connectome networks and showed that k‐clique percolation of an anomalously high order is characteristic of the human structural connectome. The resulting structural organization maintains a high local density of connectivity distributed throughout the connectome while preserving the overall sparsity of the network. To analyze these findings, we proposed a novel model for the emergence of high‐order clique percolation during network formation with a phase transition dynamic under constraints on connection length. Investigating the structural basis of functional brain subnetworks, we identified a direct relationship between their interaction and the formation of clique clusters within their structural connections. Based on these findings, we hypothesize that the percolating clique cluster serves as a distributed bridge between interacting functional subnetworks, showing the complex, complementary nature of their structural connections. We also examined the difference between individual‐specific and common structural connections and found that the latter plays a sustaining role in the connectivity of structural communities. At the same time, the superiority of individual connections, in contrast to common ones, creates variability in the interaction of functional brain subnetworks.

## Graphical abstract

Clique percolation of an anomalously high order in the structural connectome reflects the interaction of functional brain networks. The observed structural organization reveals a high local connectivity density while maintaining overall network sparsity. We propose a novel model for the emergence of clique percolation under biological constraints on connection length.

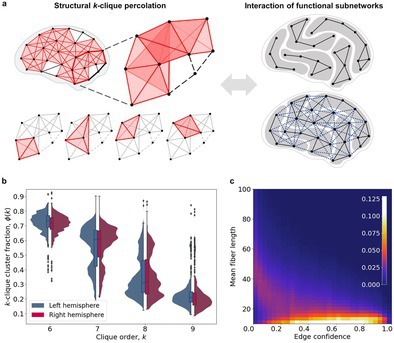

## 1 Introduction

The anatomical and functional connection networks of the human brain are increasingly studied through the lens of network science, based on graph theory techniques and terminology (Guerra et al. 2021; Szalkai et al. 2015; Zeeman 1965). This approach provides a powerful framework for understanding the complex structural organization of brain connections.

In this network representation, a structural connectome depicts the brain's anatomical connection network. Small regions of gray matter are referred to as nodes, while edges represent the axon fibers connecting these regions, as identified through diffusion MRI (Tzourio‐Mazoyer et al. 2002; Fan et al. 2016). This model allows for a comprehensive analysis of brain connectivity patterns.

Complex networks, including brain networks, often exhibit mesoscale or global structural features. Of particular interest is the community structure, characterized by densely connected node communities with sparse or weak intercommunity connections (Girvan and Newman 2002; Fortunato 2010). In the structural connectome, the intersection and interaction of these dense clusters are believed to underpin various cognitive phenomena (Bullmore and Sporns 2012; Betzel and Sporns 2016). However, the organizational principles governing the community architecture of human brain networks remain poorly understood.

Recent studies have shed light on some aspects of this architecture. For instance, study (Chang et al. 2022) demonstrated that structural connectomes exhibit a high degree of community overlap, which correlates with cognitive flexibility. This analysis employs both the Louvain community detection algorithm and the link community algorithm to uncover community overlaps in the brain. Thus, while these studies have revealed some properties of the brain's network architecture, they often fail to explain the underlying mechanisms that lead to the formation and interaction of these communities, particularly at higher levels of connectivity. For example, traditional methods do not capture higher‐order structural motifs that involve transitive closure beyond dyadic relationships.

In contrast, the k‐clique percolation method identifies overlapping communities by detecting percolation clusters of adjacent k‐cliques, where adjacency is defined by sharing $\left(k-1\right)$ nodes (Derényi et al. 2005; Palla et al. 2005). This approach, originally introduced for social and biological networks (Palla et al. 2005), reveals a distinct class of overlapping organization grounded in densely interconnected local structures. The concept of k‐clique percolation has recently gained traction in cognitive and semantic network analysis. Studies (Cosgrove et al. 2021; Valba and Gorsky 2022) have applied this method to investigate aging in semantic memory and the capacity limits of working memory, respectively. These applications demonstrate the method's potential for unraveling complex cognitive processes, particularly in cases where brain regions may contribute to multiple cognitive functions.

In this paper, we analyze the community organization of human connectomes, focusing on the dataset described in (Kerepesi et al. 2017). This dataset comprises structural connectomes of 426 human subjects, derived from the Human Connectome Project (HCP). We propose a novel rule for the connectome network evolution, which can be viewed as a modification of exponential random graphs under metric constraints. This mechanism yields clique communities of orders consistent with observed data. Our approach builds upon existing models of brain network development, such as the trade‐off between wiring cost and topological complexity (Bullmore and Sporns 2012). However, our model introduces additional constraints on connection length, reflecting the biological limitations of axonal projections in the brain. This extension allows us to more accurately capture the formation of high‐order clique structures observed in human structural connectomes while still maintaining the principles of efficient information processing and metabolic cost minimization that are fundamental to brain organization.

To analyze how the structural organization of the human brain reflects functionality, we used data on functional subnetworks (Yeo et al. 2011). Our approach to analyzing the organization of structural communities reveals a complex interplay between observed structural features and the interaction of functional subnetworks in the human brain. By investigating the relationship between these functional subnetworks and the structural features we observe, we aim to bridge the gap between structural and functional connectivity, potentially offering new insights into cognitive processes and brain disorders. For instance, recent studies have shown that alterations in structural connectivity patterns are associated with various neurological and psychiatric conditions. The study (Fornito et al. 2015) demonstrated that schizophrenia patients exhibit disruptions in the rich club organization of brain networks, which may contribute to cognitive deficits. Similarly, it was found that brain disorders target specific network hubs (Crossley et al. 2014), suggesting that our understanding of structural–functional relationships could inform targeted interventions. By analyzing the relationship between structural connectivity and functional subnetworks, our research may contribute to the development of more precise diagnostic tools and personalized treatment strategies for a range of brain disorders.

## 2 Methods

### 2.1 Data Description

*Structural connectome data*: We used high‐resolution structural connectome data from the HCP (Kerepesi et al. 2017; McNab et al. 2013), comprising 426 healthy adult participants aged 22–35. The connectomes were constructed at multiple resolution scales (83, 129, 234, 463, and 1015 nodes); for our analyses, we used the highest resolution of 1015 nodes. Each node corresponds to an anatomically defined brain region of interest (ROI), and edges represent axonal connections derived from diffusion MRI tractography.

Subcortical structures have widespread connections across the brain, often appearing as hubs in network representations. In our analysis, we identified a densely connected subnetwork comprising vertices that represent hubs and belong to subcortical structures. To focus on the broader network architecture, this densely connected subnetwork was excluded from further analyses across the entire ensemble of networks.

*Functional subnetworks data*: To analyze the relationship between structural connectivity and functional organization, we used functional subnetworks previously described in (Yeo et al. 2011). The experimental dataset consists of functional magnetic resonance imaging (fMRI) data from a large cohort of 1000 healthy young adults aged 18–35 years. The fMRI data were aligned using a common surface‐based coordinate system that preserves cortical surface topology. This dataset enables a parcellation of the human cerebral cortex based on intrinsic functional connectivity. The use of the standardized *Lausanne anatomical atlas* (Cammoun et al. 2012) allows for consistent mapping between the nodes of the structural connectome and the defined functional subnetworks. In the resulting partition, each node in the structural connectome was assigned uniquely to a functional subnetwork (Yeo et al. 2011).

We distinguished between internal and external subnetwork connections. Internal connections are edges in the structural connectome where both vertices belong to the same functional subnetwork. External connections, on the other hand, are edges in which only one vertex belongs to the given subnetwork. External connections become internal when considering the union of two or more subnetworks.

### 2.2 *k*‐Clique Percolation

**FIGURE 1:**
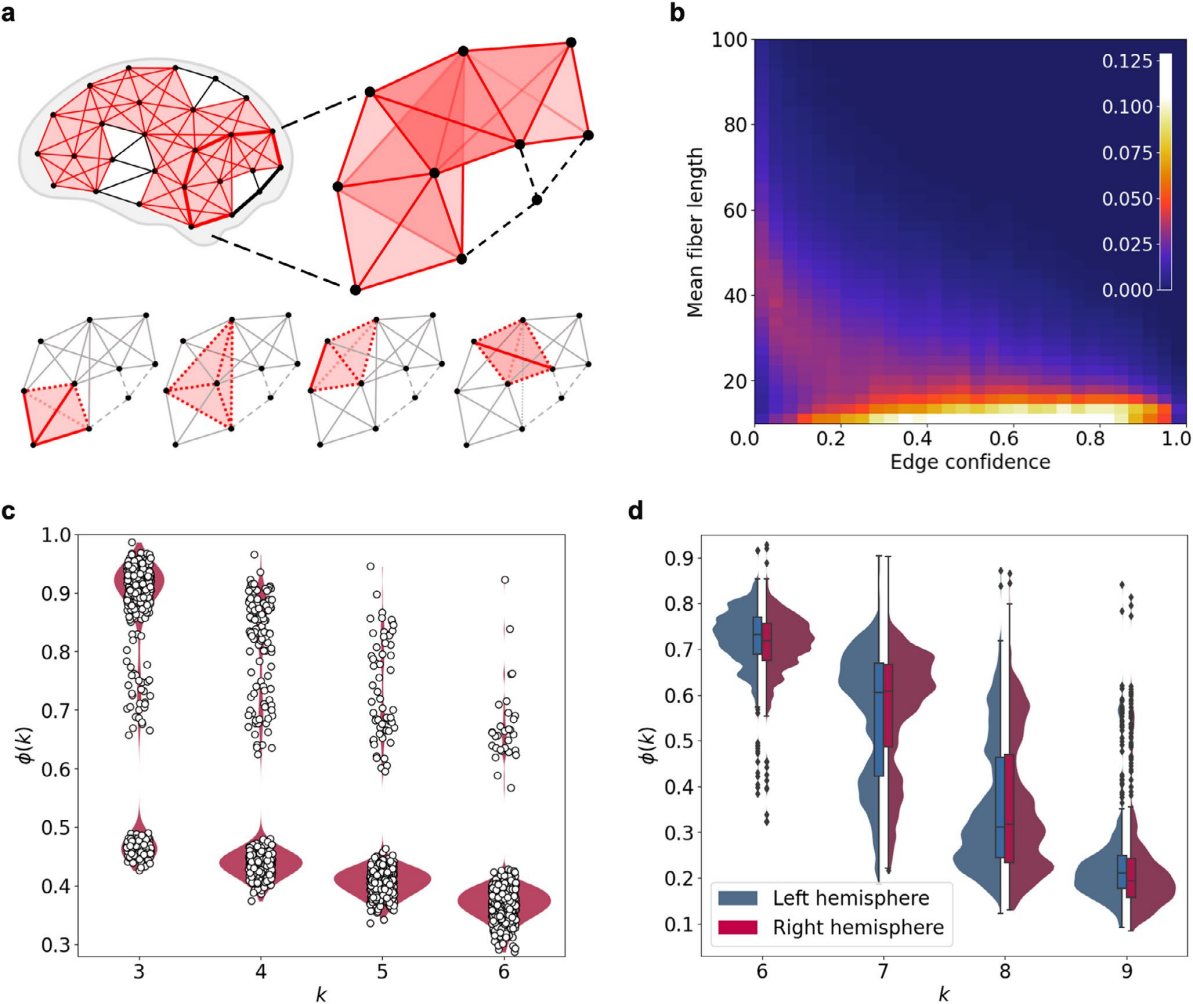
(a) The structure of the percolation k‐clique cluster occupies the entire volume of the network and consists of merged k‐cliques. (b) The distribution of structural connectome connections by the length of the forming fibers and edge confidence. (c) The fraction of the k‐clique cluster $\phi \left(k\right)$ from the entire connectome network for different clique orders k (dots represent individual structural connectomes). (d) The fraction of the clique cluster $\phi \left(k\right)$ in brain hemispheres.

A k *‐clique* is a fully connected subgraph of k nodes (Derényi et al. 2005; Palla et al. 2007). Two k‐cliques are *adjacent* if they share k−1 nodes. A k *‐clique chain* is a sequence of pairwise adjacent k‐cliques, each sharing $\left(k-1\right)$ vertices with the next. If two k‐cliques are connected by at least one such chain, they are considered k *‐clique‐connected*. Finally, a k *‐clique percolation cluster* represents the maximal k‐clique‐connected subgraph, encompassing all k‐cliques that are k‐clique‐connected to a specific k‐clique (Figure 1a).

We characterized k‐clique percolation by the order parameter $\phi \left(k\right)$, defined as the fraction of network vertices included in the largest k‐clique‐connected component. Percolation is typically said to occur when a clique cluster occupies nearly the entire network and the order parameter $\phi \left(k\right)\to 1$ (Figure S1). To benchmark empirical observations, we compared them to percolation thresholds in Erdős–Rényi random graphs. Erdős‐Rényi graphs exhibit a series of phase transitions as the probability p of connection between any two nodes increases. For k=2, this transition is well understood and manifests as the emergence of a giant component when the critical probability $p_{c}\left(k=2\right)=\frac{1}{N}$ is reached, where N denotes the number of nodes in the network. This phenomenon generalizes to higher values of k, where for each k, there exists a threshold probability $p_{c}\left(k\right)$ above which k‐cliques organize into a giant community (Palla et al. 2007):

$$p_{c}\left(k\right)=\frac{1}{{\left[N\left(k-1\right)\right]}^{1/\left(k-1\right)}}$$

This provides an analytic baseline for assessing whether high‐order percolation in empirical networks reflects non‐random organization (Bullmore and Sporns 2009).

### 2.3 The Edge Confidence

To quantify the reliability of connections across subjects, we used an edge confidence measure, similar to the methodology proposed in (Szalkai et al. 2015, 2017). The weight of an edge was calculated as its probability of occurrence across the entire set of networks. This approach helps to distinguish between common connections in the brain network and more individual‐specific ones.

### 2.4 Network Decomposition and Inverse Decomposition

To investigate how structural properties depend on edge confidence, we used two complementary approaches: network *decomposition* and *inverse decomposition*. In network decomposition, we applied a cutoff threshold τ for edge confidence, removing all links with weights below this threshold. Conversely, in inverse decomposition, we removed connections with weights greater than or equal to a threshold θ, allowing us to analyze subgraphs of weak (individual) links. The network decomposition and inverse decomposition techniques allow analyzing how different structural properties shape the organization of the connectome.

### 2.5 Network Model of High‐Order Clique Formation

We developed a generative model to examine whether high‐order k‐clique percolation can arise under anatomical constraints of human connectomes. The model can be viewed as a modification of exponential random graph models under metric constraints. We initialize the system as a random graph with 100 nodes and 1000 edges, parameters selected to approximate the average edge density observed across empirical connectomes. Each node is placed randomly but uniformly within a unit‐radius sphere in 3D Euclidean space. The positions of nodes are fixed throughout the simulation, establishing a stable anatomical embedding in which edge lengths correspond to Euclidean distances. This framework enables us to apply connection‐length constraints, reflecting the biological cost of long‐range axonal projections.

The network then evolves through an edge‐rewiring process that increases the frequency of triangular motifs, driving local clustering while preserving both the number of nodes and the total number of edges (i.e., constant network size and density). Edge rewiring was implemented as a stochastic process guided by network transitivity. At each iteration, a randomly selected edge is rewired, and the new network configuration is accepted according to one of two criteria: (1) if the new transitivity is greater than the current one (i.e., $T^{\prime }>T$); or (2) via a Metropolis‐like condition governed by a chemical potential parameter μ:

$$e^{\mu \left(T^{\prime }-T\right)}>r,\;\text{where}\;r∼U\left(0,1\right)$$

Here, transitivity T is defined as the ratio of the number of closed triads (triangles) to the number of connected triples (open triads), and $U\left(0,1\right)$ is the uniform distribution over the interval [0, 1]. The chemical potential μ thus controls the system's sensitivity to increases in transitivity: high μ favors more selective, optimal rewiring, while low μ allows greater structural exploration. If the Euclidean distance to the candidate node exceeded the threshold defining the maximum allowable connection length, a new node was selected instead. The evolution proceeds until either a predefined transitivity ceiling is reached or further increases become computationally prohibitive. In our simulations, the maximum transitivity is capped at 0.6, slightly above the empirical mean of ∼0.5 in human connectomes.

In our model, low chemical potential corresponds to a high probability of edge rewiring regardless of the contribution to transitivity, that is, the acceptance of non‐optimal states (contrary to typical optimization problems due to the sign of the transitivity difference). Under these conditions, edge rewiring occurs almost indiscriminately, regardless of changes in transitivity, resulting in the generation of a new random graph on a fixed set of vertices at nearly every step. Thus, we can analytically estimate the expected maximal order of the percolation cluster $\rho_{c}$ for such random graphs.

### 2.6 Network Embedding

We applied two‐dimensional hyperbolic embedding to project structural networks into a geometric space, allowing analysis of latent similarity and hierarchical relationships between nodes (Allard and Serrano 2020; Whi et al. 2022; Zheng et al. 2020; Tadić et al. 2019). Each node received a radial coordinate (degree‐based centrality) and an angular coordinate (structural similarity).

For embedding, connections with edge confidence less than 0.2 were discarded to emphasize common, robust structure across connectomes. We used the method from (Ciucci et al. 2017) to compute minimal distortion embeddings in two‐dimensional hyperbolic space to ensure that the common structure between all connectomes had a greater influence during the embedding process.

## 3 Results

We study the internal connectivity of human structural connectomes and its relation to functional subnetworks, presenting our findings as follows. We start by showing that high‐order k‐clique percolation is a distinctive feature of the human structural connectome. To understand the observed phenomenon, we proposed a novel model for emerging high‐order k‐clique percolation with phase‐transition dynamics under specific constraints on connection length. We then examined the structural connections underlying known functional subnetworks of the brain, showing how the interaction between functional subnetworks related to the formation of structural clique communities. In the last section, we explored the differences between individual and common structural connections, employing network *decomposition* and *inverse decomposition* techniques (see Section 2 for details).

### 3.1 Community Structure and k‐Clique Percolation in Human Structural Connectomes

*Higher‐order clique percolation in hemispheric networks*: In random graphs, high‐order k‐clique percolation typically requires high edge density. The observed hemispheric connection density in connectomes (i.e., the proportion of actual connections relative to all possible connections) falls within the range $p_{c}\left(k=3\right)<\rho <p_{c}\left(k=4\right)$ (see Section 2 for details). It means that the density is low that only 4‐clique percolation could occur, if the network were random. Moreover, known models typically assume the possibility of long‐range connections, whereas in connectomes we observe the opposite: the vast majority of connections are short‐range (Figure 1b, edge confidence shows the frequency of connection occurrence in the network sample).

We evaluated the presence of k‐clique percolation by computing the order parameter $\phi \left(k\right)$ (see Section 2 for the details). The observed bimodal patterns in $\phi \left(k\right)$ for certain k values arise from two distinct structural phenomena in the connectome data. First, the bump at k=3 in Figure 1c, which shows $\phi \left(k\right)$ for the entire brain, reflects interhemispheric separation. In several connectomes, triangle‐based percolation (k=3) does not span both hemispheres, likely due to the relative sparsity and low transitivity of interhemispheric connections. This leads to the formation of two large, but disconnected, percolation clusters—one in each hemisphere—resulting in a reduced overall $\phi \left(k\right)$ value compared to the within‐hemisphere case.

Second, the bumps at k=7 and k=8 in Figure 1d, which displays $\phi \left(k\right)$ for individual hemispheres, indicate the presence of dense substructures within hemispheric networks. As clique order increases, percolation becomes more selective and $\phi \left(k\right)$ typically decreases. However, the transient rise in $\phi \left(k\right)$ at k=7,8 in some connectomes suggests the existence of localized regions with exceptionally high transitivity that still support high‐order clique connectivity. These substructures do not span the full hemisphere but remain sizable enough to produce local maxima in $\phi \left(k\right)$. Thus, clique percolation analysis detects mesoscale structural patterns that would be missed by global measures.

**FIGURE 2:**
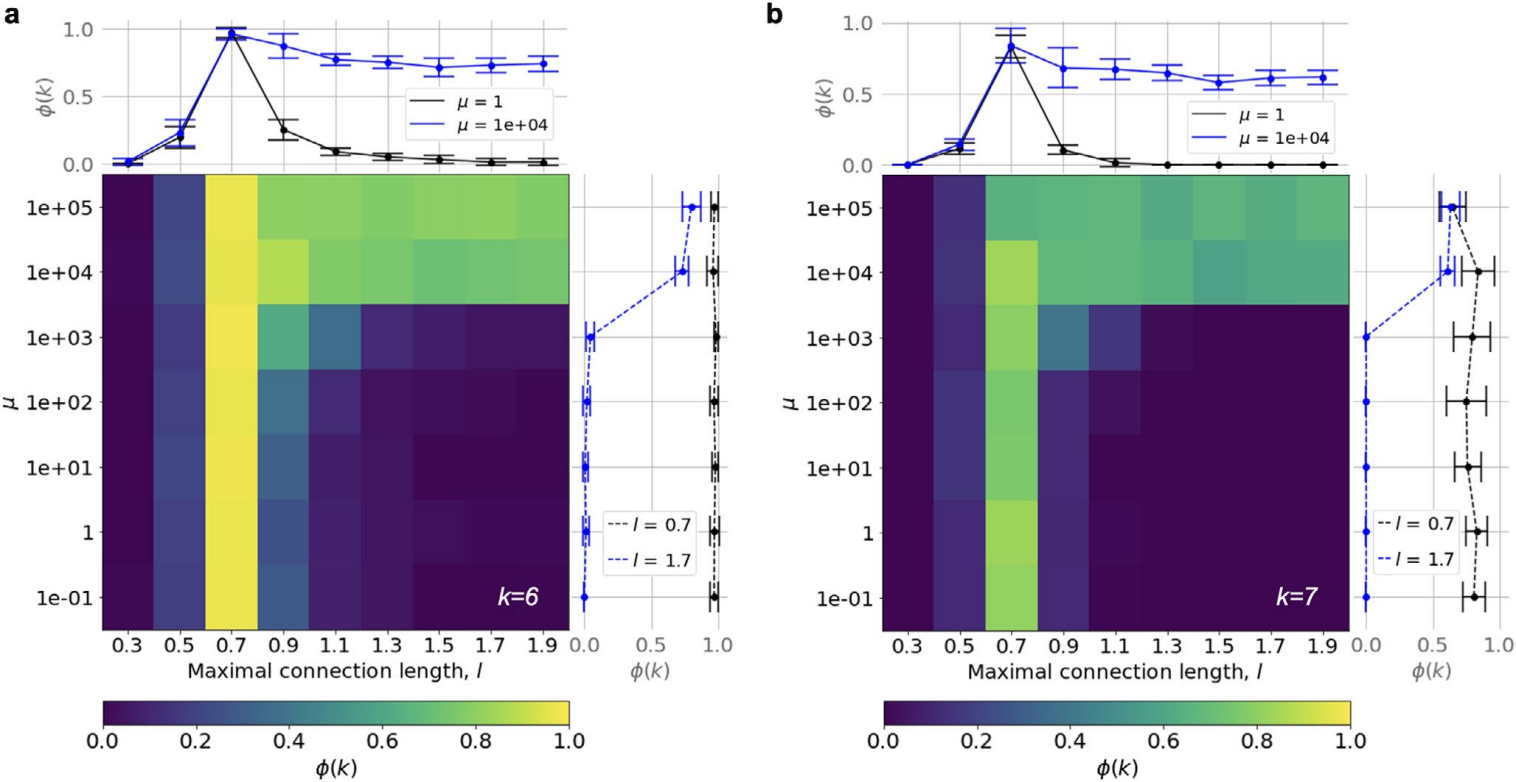
k‐clique cluster fraction of the model networks $\phi \left(k\right)$ for clique orders k=6 (a) and k=7 (b), depending on model parameters (each value is averaged over 10 networks). The networks were generated through an evolutionary process that optimizes the growth of recurrent connections under constraints on permissible connection length (*maximal connection length*) and a fixed value of the chemical potential μ. Large‐order k‐clique percolation, characteristic of human structural connectomes, occurs in a certain parametric domain. The side graphs show $\phi \left(k\right)$ change for fixed values of one of the parameters.

*A novel network model for high‐order* k *‐clique percolation in the human structural connectome*: Human structural connectomes exhibit a predominance of short‐range connections, especially among edges with high confidence values (Figure 1b). However, the overall connection density is relatively low, falling below the threshold required for high‐order k‐clique percolation in known random graph models. This raises a discrepancy: despite low global density, empirical connectomes exhibit dense, high‐order clique structures distributed throughout the network volume. To reconcile this, we developed a generative network model that explicitly incorporates spatial constraints on connection length. By simulating network evolution within a bounded spatial domain, the model enables us to examine how anatomical wiring constraints can give rise to densely connected communities and support high‐order clique percolation. Using the generative model described in Section 2, we simulated network evolution under spatial constraints and monitored the emergence of k‐clique percolation via $\phi \left(k\right)$ for each parameter setting (chemical potential μ and maximal connection length) (Figure 2).

The density of connections in the model networks, as in empirical connectomes, suggests the formation of k‐clique clusters of maximum order only up to k=3 or k=4. Remarkably, within a specific range of connection lengths, we observe persistent k‐clique percolation up to k=7 across all values of the chemical potential (Figure 2a,b; maximal edge length ≈0.7).

In our model, network structure emerges through random edge rewiring and the gradual formation of recurrent connections—processes that correspond to triangle saturation and the increase of transitivity to levels observed in connectomes. By adjusting the chemical potential during network evolution, we control how efficiently and optimally transitivity increases. Low values of chemical potential correspond to a high probability of edge rewiring, regardless of whether transitivity is improved. As a result, the network retains a largely random structure, for which we can analytically estimate the expected maximal order of the percolation cluster ρ. Indeed, under low chemical potentials and in the absence of strict connection length constraints, no percolating cluster emerges.

**FIGURE 3:**
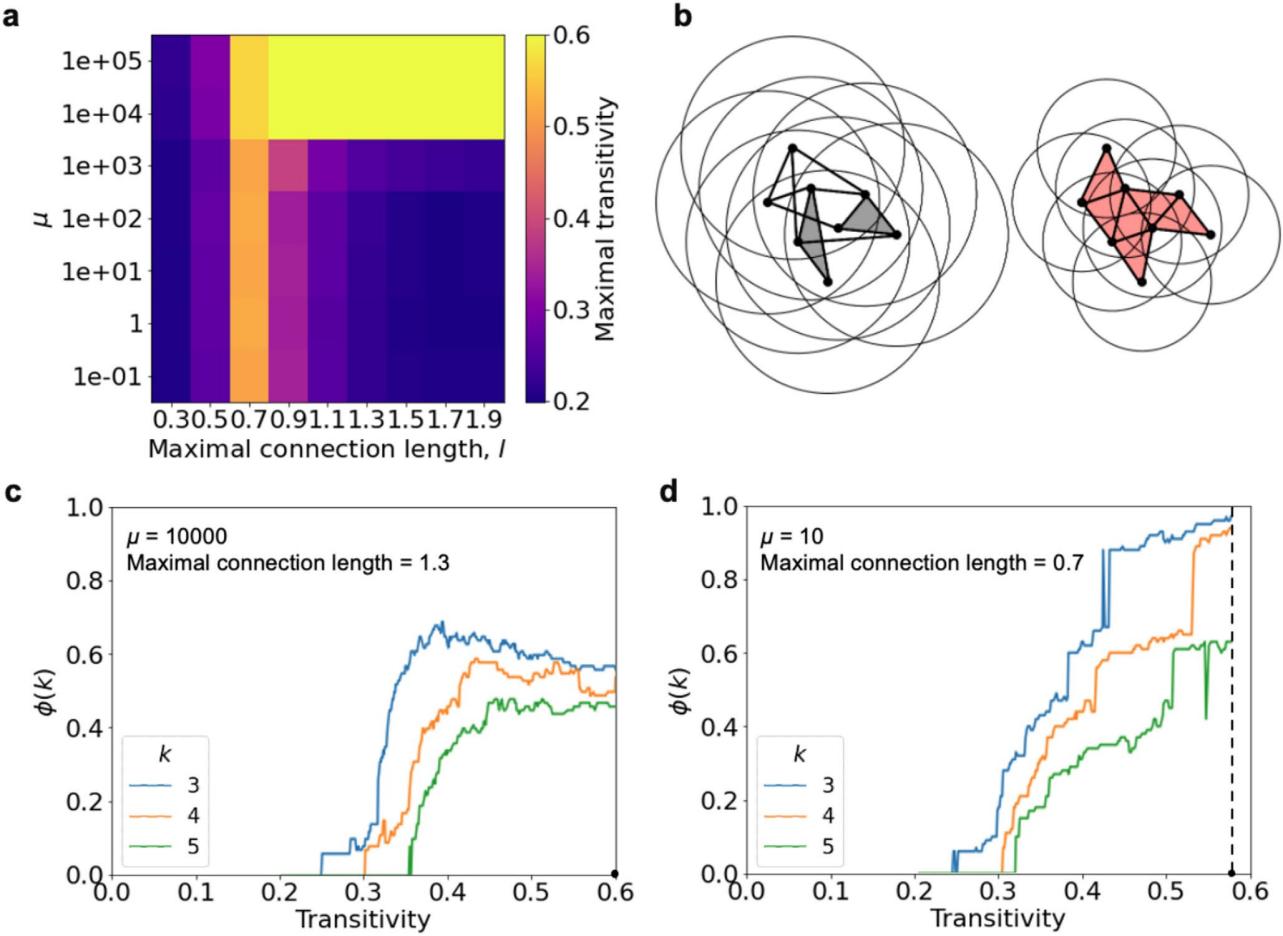
(a) The maximal value of transitivity when forming a network depends on the model parameters (each value is averaged over 10 networks). (b) Schematic illustration of the change in the network structure during random edge rewiring with different permissible connection lengths. The resulting triangles (k‐cliques with k=3) are shaded in gray. When connection lengths are sufficiently localized, a greater number of triangles form, leading to the emergence of a single percolating clique cluster (shaded in red, right panel). (c, d) Characteristic dynamics of the k‐clique clusters fraction $\phi \left(k\right)$ during model network formation with increasing transitivity. (c) At high chemical potentials, where rewired connections predominantly increase transitivity, the network tends to form dense clusters that are not necessarily interconnected. (d) When the length of connections is limited to a certain range, a percolation cluster is formed even at low chemical potential.

However, when the connection length is restricted to a specific range (≈0.7), percolation occurs, as illustrated schematically in Figure 3b. Thus, metric constraints on connection length effectively regulate local connection density, making it sufficient to support the formation of clique clusters.

At high chemical potentials, where rewired connections predominantly increase transitivity, the network tends to form dense clusters that are not necessarily interconnected. The global optimum of such dynamics is a clustered configuration in which clusters occupy only a fraction of the network volume and are tightly packed (Figure 3c). As a result, k‐cliques fail to percolate for large k, even though network transitivity reaches its maximum values (Figure 3a,c). Nevertheless, when constraints on connection length are imposed (≈0.7), clique percolation can still occur (Figure 3d). Thus, metric constraints also limit the local connection density, preventing the formation of overly dense structures and leading to the emergence of a distributed clique cluster.

### 3.2 Clique Community Organization Reveals an Interaction Between Functional Brain Subnetworks at the Level of Structural Connections

*High structural intertwining of functional subnetworks*: Recent research revealed several functionally connected subnetworks within human functional connectomes, characterized by a high degree of separability (i.e., reproducibly distinguishable based on their connectivity patterns) (Yeo et al. 2011). We studied the structural connections underlying these functionally separated subnetworks using the same anatomical parcellation (Cammoun et al. 2012).

**FIGURE 4:**
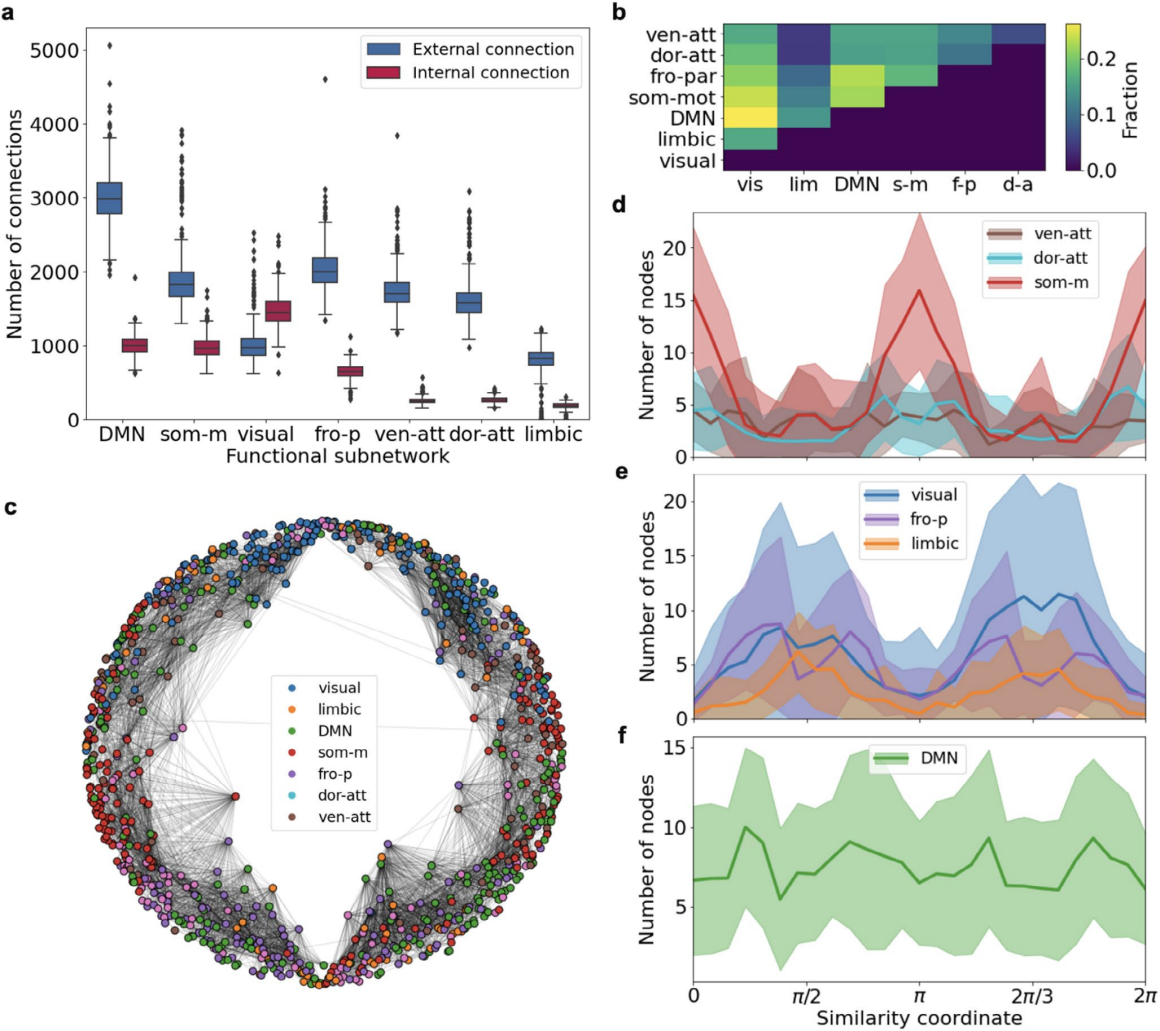
(a) The number of external and internal structural connections of functional subnetworks of the human brain. (b) The fraction of external connections connecting functional subnetworks in pairs (each value is averaged over the entire sample of connectomes). (c) Hyperbolic embedding of the structural connectome allows studying the similarity between nodes based solely on the structural properties of the network. Colors of nodes correspond to different functional subnetworks. (d–f) Distribution of connectome nodes along similarity coordinate for different functional subnetworks: *Ventral‐attention, dorsal‐attention, somato‐motor* (d), *visual, fronto‐parietal, limbic* (e), and *default mode network (DMN)* (f).

In all subnetworks except the *visual* network, external connections outnumbered internal ones (Figure 4a). This trend was most pronounced in smaller subnetworks.

Analysis of subnetwork connections showed that external connectivity was broadly distributed: the proportion of connections between any two subnetworks was low and relatively uniform (Figure 4b).

Given such dense interaction of subnetworks, we next explored the node's functional proximity based solely on the structural organization and compared it in the context of belonging to one of the functional subnetworks. It has recently been shown that human connectomes can be anatomically analyzed using hyperbolic embedding (Allard and Serrano 2020; Whi et al. 2022; Zheng et al. 2020; Tadić et al. 2019).

Using hyperbolic embedding, we mapped each connectome into a two‐dimensional space defined by similarity and centrality coordinates. Figure 4c shows the embedding for one of the connectomes. Each node acquires two coordinates in a two‐dimensional hyperbolic embedding, where the radial coordinate is related to the node hubness, and the second coordinate can be used as a measure of node similarity. The node's colors correspond to the functional subnetworks to which they belong, and the clustering of nodes by color along the bridge of the embedding is visually distinguishable.

Observing the distribution of nodes of each functional subnetwork by the similarity coordinate, we observe the complexly mutual complementary patterns (Figure 4d–f). Except for the *default mode network (DMN)*, two groups can be roughly distinguished among all functional subnetworks by the similarity of the distribution pattern (Figure 4d,e). In each group, the distribution pattern is periodic, clearly reflecting the hemispheres' separation. Interestingly, within each hemisphere, the structure is reproduced within the sample of networks and, in a sense, complements each other due to the coincidence of density bumps according to the similarity. The *DMN* network, on the contrary, is distributed more evenly, merged with all other subnetworks at once, and ambiguously relates to each of the previously identified groups (Figure 4f). This finding aligns with previous research emphasizing how the *DMN* supports information integration across various brain regions (Raichle 2015). The intersection of similarity profiles across different subnetworks reveals their complex interweaving, preventing a clear structural separation based on a single‐dimensional similarity measure (Figure 4c–f). It is possible that better separation could be achieved in higher‐dimensional embeddings, where similarity is evaluated along multiple axes simultaneously, which is beyond the scope of the present study. As a result, we observe the grouping of known functional subnetworks by a completely different method, based solely on the internal structure of connections.

**FIGURE 5:**
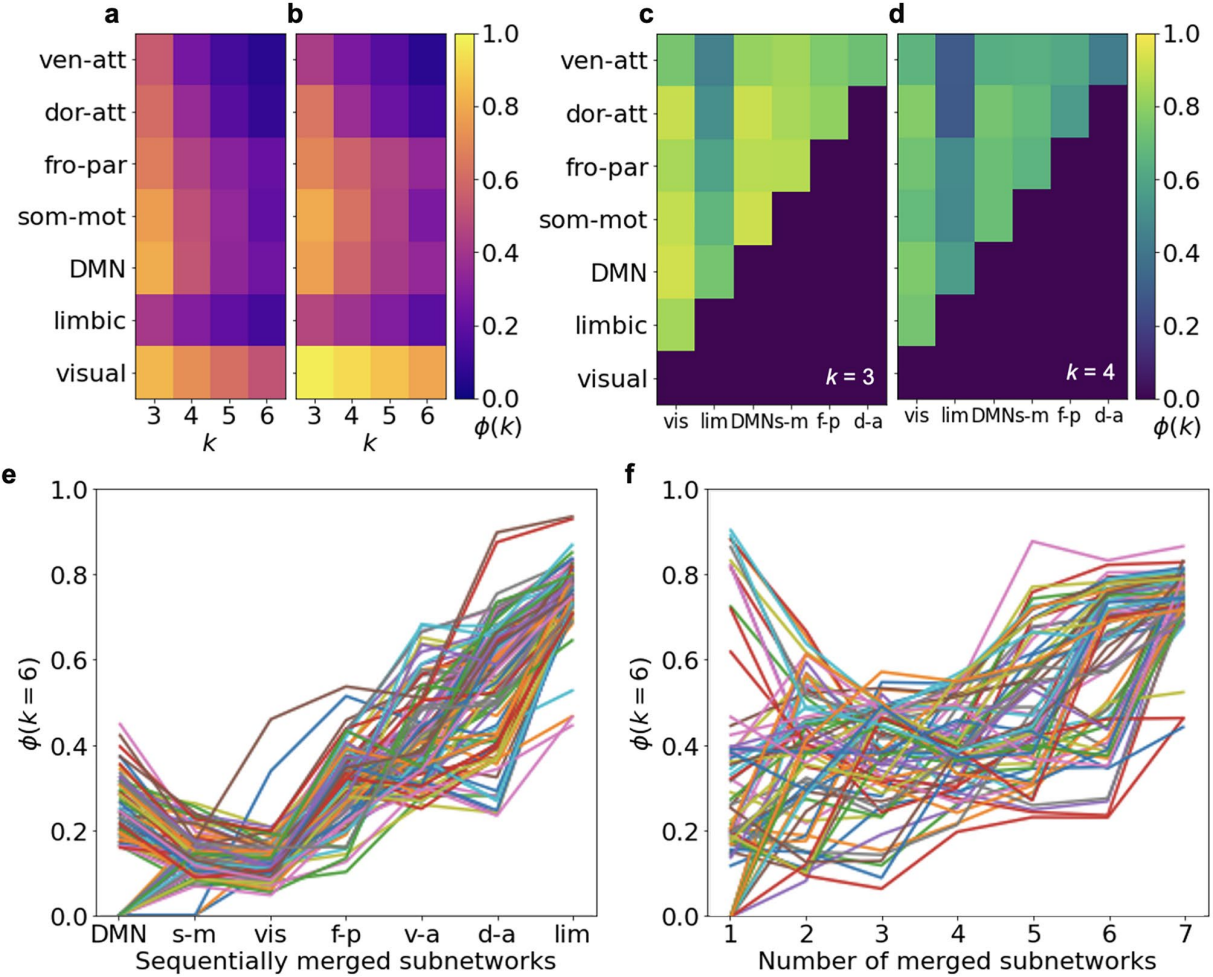
Analysis of k‐clique clusters in functional subnetworks and their interactions. (a, b) Proportion of k‐clique clusters in individual subnetworks: (a) considering only internal connections and (b) including both internal and external connections. (c, d) Proportion of k‐clique clusters resulting from pairwise merging of subnetworks for clique orders (c) k=3 and (d) k=4. Values in (a–d) represent averages across 100 networks. (e, f) Growth dynamics of k‐clique cluster fraction $\phi \left(k\right)$ during sequential subnetwork merging: (e) ordered by subnetwork size and (f) in random order. Each curve corresponds to one connectome from the dataset (the plots show changes in $\phi \left(k\right)$ during the merging of subnetworks for 100 randomly selected connectomes). Despite individual variability, the fraction occupied by the clique percolation cluster increases almost linearly with the addition of each new subnetwork.

*The emergence of high‐order clique structures requires simultaneous interaction of several functional subnetworks*: Next, we investigated the clique structures in functional subnetworks. When examining each subnetwork in isolation, the maximum order of clique clusters with sufficient fraction does not exceed k=3−4, regardless of whether external connections are included (Figure 5a,b).

The highest proportion of high‐order clique structures is observed in the *visual, DMN, somatomotor*, and *frontoparietal* subnetworks (Figure 5a,b), which is directly related to the number of connections in these subnetworks.

When considering pairwise unions of subnetworks (including only internal connections for united pairs), the *DMN* and *somatomotor* networks demonstrate the most complementary connectivity with other subnetworks through clique structures (Figure 5c,d). For most combinations involving the *DMN*, the resulting fraction of k‐clique clusters exceeds the initial values for both the *DMN* and the other subnetworks involved.

We then analyzed the sequential merging of all subnetworks and tracked $\phi \left(k=6\right)$ across steps. At each step, $\phi \left(k\right)$ represents the proportion of vertices in the k‐clique cluster relative to all vertices in the already combined subnetworks, not the entire connectome. Both size‐based (Figure 5e) and random merging orders (Figure 5f) showed near‐linear growth of the percolation cluster. High‐order clique percolation was achieved only after integration of all subnetworks. These findings show that the complementarity of external connections, i.e., the interaction of subnetworks, maintains the high‐order clique percolation cluster.

### 3.3 High‐Confidence Connections Sustain High‐Order Clique Percolation

*Edge confidence is associated with the sustainability of community structure connectivity in the human structural connectome*: To assess the structural basis of clique cluster stability, we applied decomposition and inverse decomposition based on edge confidence thresholds (τ and θ, respectively; see Section 2 for details).

**FIGURE 6:**
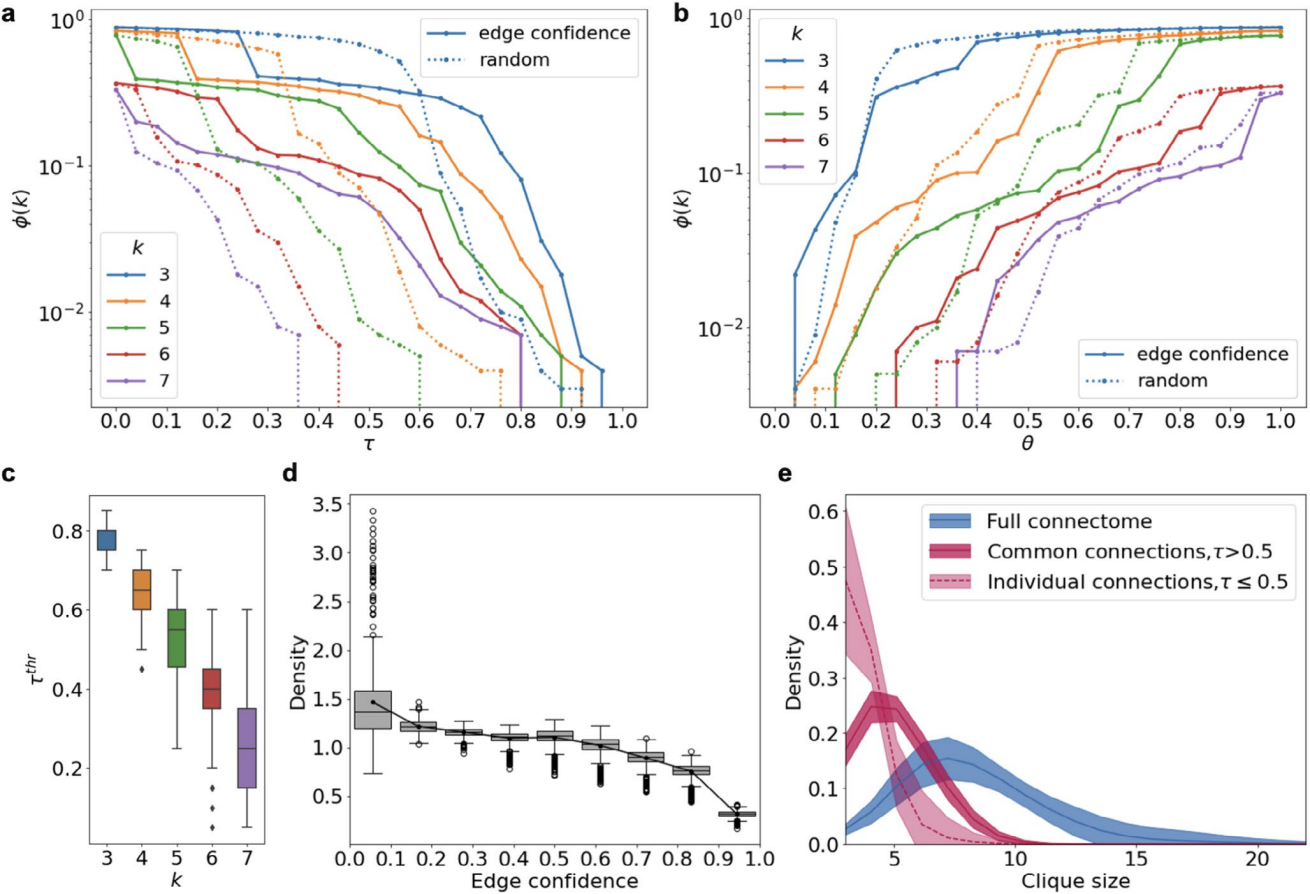
(a, b) The fraction of nodes ($\phi \left(k\right)$) included in k‐clique clusters as a function of the cutoff threshold (a) τ for network decomposition and (b) threshold θ for inverse decomposition. (c) Distributions of critical destruction thresholds for clique clusters across structural connectomes, shown for different clique orders k. (d) Distribution of edge confidence values in individual human structural connectomes. (e) Distributions of maximal clique sizes in connectome networks.

In decomposition, higher‐order k‐clique clusters (k=5,6,7) degraded rapidly as τ increased from 0 to 0.2. In contrast, $\phi \left(k=3\right)$ and $\phi \left(k=4\right)$ remained high until τ≈0.4−0.5 (Figure 6a). The interhemispheric binding structure for k=3 clusters disintegrates at τ≈0.2. This behavior aligns with density‐dependent k‐clique percolation observed in random network models (see Section 2, k‐clique percolation section). Randomized edge removal (dotted lines) resulted in faster loss of connectivity across all k. In inverse decomposition, for k≥4, $\phi \left(k\right)$ increased only at large τ values, corresponding to the addition of high‐confidence connections (Figure 6b). Random edge addition restored clique clusters more quickly than confidence‐based addition.

Distributions of critical destruction thresholds (peak derivative points of $\phi \left(k\right)$) across networks are shown in Figure 6c. As the order k increases, the spread of the destruction threshold increases, indicating heterogeneity in the dense community structures within human connectomes. The overall distribution of edge confidence values is shown in Figure 6d. The cutting occurs according to the edge confidence values, which are distributed in individual networks quite evenly, excepting the extremely small and large values (Figure 6d). The peak in the distribution of the lightest connections is most likely associated with errors in their determination, and they are also distinguished when studying the characteristic length of connections, constituting the overwhelming majority of the long‐range domain (Figure 1b).

The observed difference between individual and common connections in importance for the clique structures connectivity is also confirmed by observation of the distributions of maximum cliques. In the initial network state, the characteristic clique size ranges within 5−10 and reaches 20 in the heavy right tail of the distribution (Figure 6e). We compared the distribution of maximal cliques if we cut half of the most individual or, on the contrary, half of the most common connections in connectomes. Comparison of the distribution shifts shows the difference between the influence of individual and common connections on the characteristic clique sizes. Note that the effect is present despite the fact that more connections remain in the network when cutting half with higher edge confidence values.

**FIGURE 7:**
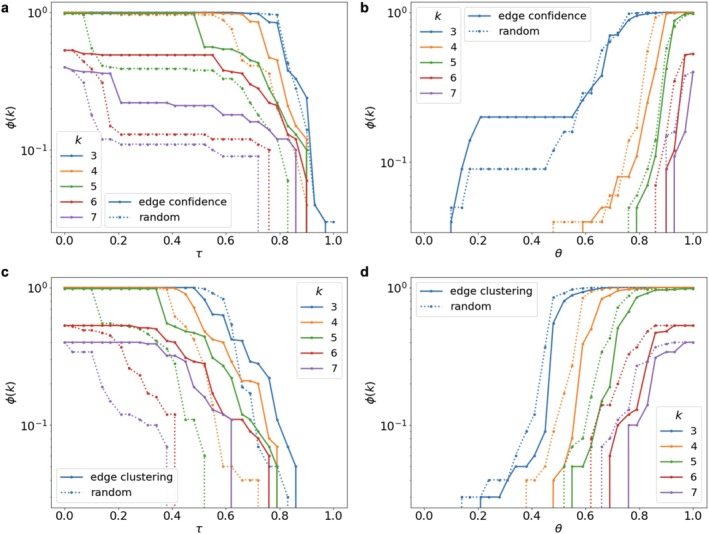
Model network decomposition (a, c) and inverse decomposition (d, b) using (a, b) generated sample‐based edge confidence and (c, d) edge clustering coefficient weights. Solid lines represent the k‐clique cluster size when edges are removed or added based on their weight; dotted lines show the cluster size when edges are randomly removed or added in the same proportion.

Then, we calculated edge confidence for a sample of model networks. These networks were generated through the previously described process, increasing their transitivity to 0.4 under fixed connection length constraints and with fixed node coordinates (the maximum connection length was set to 0.7 in each of 200 model networks with 100 nodes and 1000 edges). For both decomposition and inverse decomposition processes in model networks, edge confidence has a similar relation to the stability of clique cluster connectivity (Figure 7a,b).

Network destruction also occurs later when compared to random edge removal in decomposition. During inverse decomposition, the cluster growth dynamics mirror those in connectomes; the earlier addition of heavy edges due to random selection leads to faster cluster volume recovery. Additionally, we explored another connection characteristic, the edge clustering coefficient, which represents the proportion of potential triangles associated with each edge, ranging from 0 to 1 (Valba and Gorsky 2022) (Figure 7c,d). While the critical thresholds and the dynamics of network destruction and restoration differ due to variations in the weight distributions across network edges, the effect related to sustainability of decomposition is consistent for both model weights.

**FIGURE 8:**
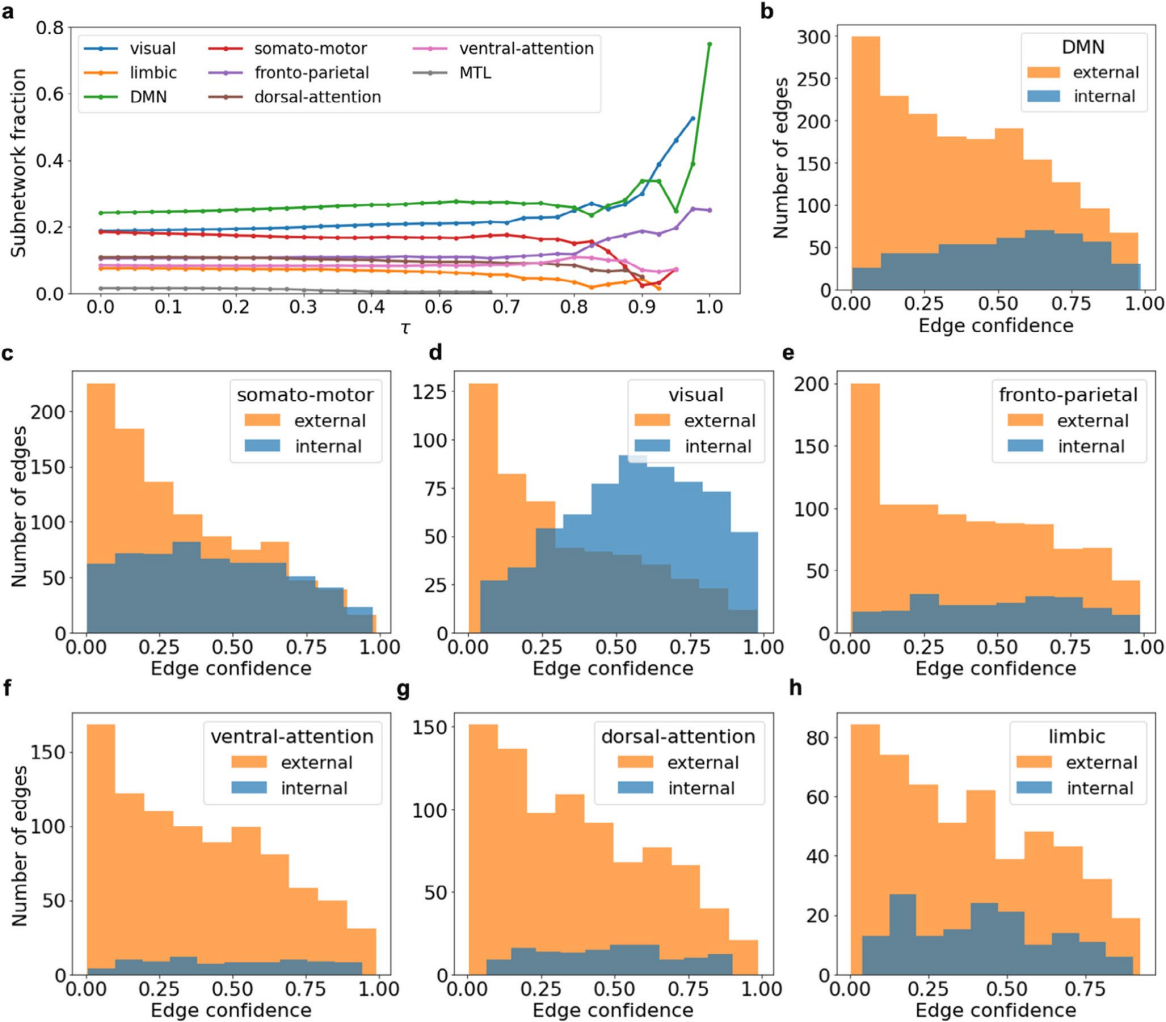
(a) The fraction made up of subnetwork nodes during decomposition by edge confidence value. An individual node is no longer taken into account if there are no connected edges of the subnetwork left. (b–h) Distributions of edge confidence for external and internal connections of subnetworks.

During the decomposition process, subnetworks retain their relative fraction until the onset of critical collapse, when only the most common connections remain and the network loses connectivity (Figure 8a). The threshold for the onset of critical destruction of subnetworks is in the range at which *k* = 3–4‐clique percolation clusters disintegrate in individual connectomes and random graphs of equivalent density. This finding demonstrates the robustness of functional subnetworks up to a critical point.

*Structural connections of interaction between functional subnetworks are more diverse and individual than internal ones*: Functional subnetworks differ significantly not only in the number of external and internal connections but also in their characteristic edge confidence distributions (Figure 8b–h). The peak in the distribution of edge confidence in connectomes in the domain of the most individual connections turns out to be formed by external connections of subnetworks. Internal connections are more evenly distributed by the edge confidence parameter, showing a greater commonality in the internal structure of functional subnetworks in comparison with the individual diversity of their external connections. The *visual* subnetwork stands out as the only one with more internal than external connections, with its internal connections showing a bias towards higher edge confidence values (Figure 8d). This may reflect the specialized and highly integrated nature of visual processing in the brain (Bullmore and Sporns 2009).

## 4 Discussion

*The brain's structural connectome balances efficiency and constraints through high‐order clique clusters, providing dense local connectivity in distributed brain networks*: Using network science approaches, we demonstrated clique percolation of remarkably high order in human connectome networks. These structures reflect dense, highly overlapped structural communities distributed throughout the connectome. They also form distributed bridges between interacting functional subnetworks, demonstrating a complex organization of brain connectivity. We suggest that these clusters emerge as a result of metric constraints on connection length, reflecting the brain's evolutionary need to balance connectivity efficiency with biological limitations. The observed order of the percolating clique cluster is anomalous for the characteristic connectome density and could not be explained as a density‐dependent phenomenon.

The resulting structural organization maintains a high density of local connectivity distributed across the connectome while preserving overall network sparsity. These high‐order structures emerge at relatively low global connection densities—an efficient architecture consistent with known constraints on metabolic cost and wiring length (Bullmore and Sporns 2012). A certain level of local connectivity density may be critical for the emergence of dynamic attractors at the level of neural circuits, such as specific synchronization regimes or activity localization, particularly bump attractors (Hansel and Sompolinsky 1998; Tiselko et al. 2023). The presence of large, structurally overlapping k‐clique clusters distributed across the brain may also reflect an organizational principle that balances local specialization with global accessibility. This aligns with prior findings that brain networks exhibit small‐world and rich‐club properties, characterized by high clustering and short path lengths that support both functional segregation and integration (Watts and Strogatz 1998; Van Den Heuvel and Sporns 2011).

Our new network model, incorporating physiological constraints on connection length, successfully replicated the observed structural properties. This model is based on the dynamical formation of the network structure through a process of random connection rewiring, increasing recurrent connections while maintaining network density. Length constraints resemble those in geometric graph models, where connectivity depends on spatial distance; however, in our model, such constraints act dynamically during structural evolution. By growing recurrent connections, we restore the naturally observed triangle density, reflecting recurrent connectivity in connectomes. With certain restrictions on the connection length, we observe a structural phase transition, with the emergence of high‐order k‐cliques percolation. Such a dynamic is typically associated with critical phenomena and characterized by a continuous change in the order parameter (in this case, the size of the clique percolation cluster) as the control parameter is varied (connection length constraint). However, a more detailed analysis of this phenomenon is beyond the scope of the present research and requires further investigation to fully characterize its properties and implications for brain network organization. Without restrictions on connection length, the model demonstrates how the saturation of the network with triangles leads to excessive clustering, where dense structures occupy only a fraction of the network, reaching maximal density.

*The observed structural clique clusters form a distributed structural bridge between interacting functional subnetworks*: We examined the structural basis of functional brain subnetworks, which are known to exhibit high separability and reproducibility in functional connectomes (Yeo et al. 2011). We find that high‐order k‐clique clusters do not form within isolated functional subnetworks; instead, they only emerge when multiple subnetworks are merged, suggesting that their formation requires extensive inter‐subnetwork connectivity. Functionally separable subnetworks do not correspond to well‐defined structural clusters and possess more external than internal structural connections. These external connections are shown to be more variable and individual‐specific, which may indicate a role in flexible integration. Taken together, these findings support the hypothesis that high‐order percolating cliques form a shared structural substrate across interacting subnetworks, reflecting the mutually complementary nature of structural organization and complex interplay between functional and structural networks, which enables structural integration without strict modular segregation. While we do not claim direct causality, the convergence of these structural features suggests a potentially fundamental role for clique percolation in shaping the mesoscale architecture of brain connectivity.

It was proposed that high‐order cliques in structural brain networks could serve as building blocks for cognitive processes, potentially Supporting Information integration across different brain regions (Sizemore et al. 2019). The existence of these structures might contribute to the brain's resilience and adaptability (Bullmore and Sporns 2012). This finding aligns with recent research suggesting that brain networks exhibit small‐world properties and rich‐club organization, facilitating efficient information processing and integration (Watts and Strogatz 1998; Van Den Heuvel and Sporns 2011).

*High‐confidence, population‐common connections drive clique formation and sustain a reproducible structural backbone despite individual connectome variability*: By analyzing the difference between individual and common connections for connectomes from the sample, we observed the distinguished role of the latter in the formation and connectivity of clique communities. The edge clustering coefficient, which is another connection characteristic, had a similar effect, revealing a straightforward property: connections that contribute most significantly to local triangle density have the greatest impact on the formation of large cliques and maintaining connectivity within the clique communities. Another noteworthy finding is the difference in edge confidence distributions between internal and external connections of subnetworks. External connections lack the characteristic growth in distribution as edge confidence weakens; instead, their distribution is nearly uniform. This suggests that internal connections are more repetitive and common across the connectome sample, regardless of their numerical inferiority to external connections.

The consistent role of high‐confidence connections in sustaining clique organization across subjects also reflects a broader principle of structural regularity: while individual connectomes may vary, the recurrence of specific structural motifs across a population yields a stable and shared architectural core. This suggests a unifying feature of human structural connectomes, whereby interindividual variability can coexist with a reproducible backbone that supports network‐level integration.

*Implications for brain disease research and clinical applications*: Alterations in the structural connectome are increasingly implicated in a wide range of neurological and psychiatric conditions, including schizophrenia, Alzheimer's disease, autism spectrum disorder, and traumatic brain injury (Fornito et al. 2015; Crossley et al. 2014; Dennis and Thompson 2014). Our k‐clique percolation framework can serve as a powerful tool to detect disruptions in high‐order structural motifs that underlie integrative brain function. For instance, a breakdown in the integrity or percolation of large k‐clique clusters may reflect impaired communication across functional subnetworks—a phenomenon observed in disorders characterized by cognitive fragmentation or network‐level dysregulation (van den Heuvel et al. 2010; Sheline and Raichle 2013).

Additionally, the edge confidence analysis enables differentiation between stable, population‐level (common) structural backbones and more variable, individual‐specific connections. Disease‐related changes might selectively impact these individual or common structures, potentially allowing k‐clique metrics to serve as sensitive biomarkers of pathology progression or treatment response (Griffa et al. 2013). Future work could leverage this framework to identify disease‐specific patterns of clique disintegration or reorganization, contributing to early diagnosis and personalized therapeutic strategies (Bassett and Sporns 2017).

Moreover, the ability to model clique formation dynamics under biologically plausible constraints opens the door to computational phenotyping of pathological networks. By simulating how disease‐related changes in connection length, density, or transitivity affect percolation thresholds, it is possible to construct in silico models of diseased connectomes that can be compared to empirical patient data (Zimmermann et al. 2018).

## 5 Conclusion

The k‐clique percolation approach provides a powerful tool for unraveling relationships between structural and functional brain networks. The discovery of high‐order k‐cliques organization in the human structural connectome expands our understanding of brain connectivity. These complex structures support the hypothesis that the brain is not merely a collection of isolated functional modules but rather an intricately connected system where densely interlinked regions support cognitive processes across multiple domains. This finding demonstrates that higher‐order clique communities maintain both local and global network integration, potentially supporting critical cognitive functions.

## Supporting information


**Figure S1:** Schematic illustration of *k*‐clique percolation and cluster formation. (A) Disconnected 3‐cliques form isolated units below the percolation threshold. (B) Overlapping cliques begin forming chains via shared (*k*1)‐node overlaps. (C) A giant percolation cluster spans much of the network when the threshold is exceeded. (D) A phase transition in *ϕ*(*k*), the fraction of nodes in the percolation cluster emerges as edge density increases, indicating the critical threshold *p*_*c*_(*k*) for percolation.

## Data Availability

Data sharing not applicable to this article as no datasets were generated or analysed during the current study.
