# Supplementary material for: Interaction of Functional Brain Networks Is Associated With k‐Clique Percolation in the Human Structural Connectome
Source: Hum Brain Mapp. 2025 Oct 23;46(15):e70343. doi: 10.1002/hbm.70343 (PMC12547843; doi:10.1002/hbm.70343)
Supplement: Supplementary file 1 — Figure S1: Schematic illustration of k‐clique percolation and cluster formation. (A) Disconnected 3‐cliques form isolated units below the percolation threshold. (B) Overlapping cliques begin forming chains via shared (k1)‐node overlaps. (C) A giant percolation cluster spans much of the network when the threshold is exceeded. (D) A phase transition in ϕ(k), the fraction of nodes in the percolation cluster emerges as edge density increases, indicating the critical threshold p c (k) for percolation. [file HBM-46-e70343-s001.pdf]

Supplementary material

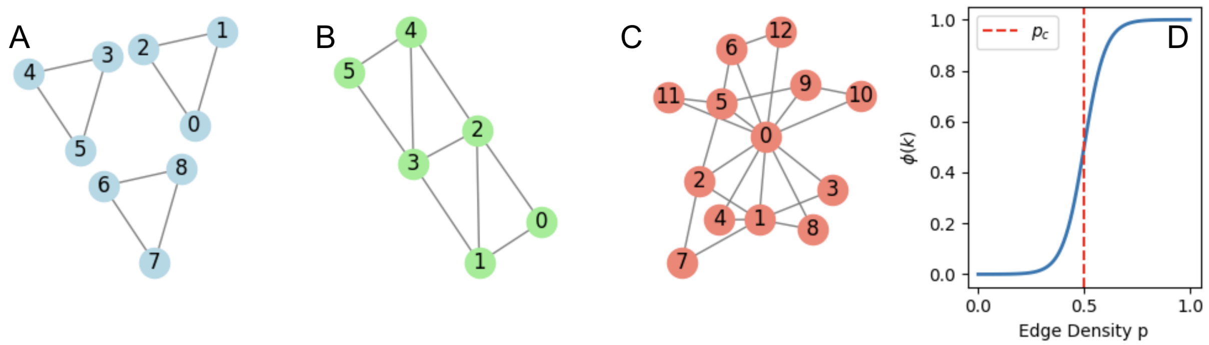

Figure 1: Schematic illustration of k-clique percolation and cluster formation. (A) Disconnected 3-cliques form isolated units below the percolation threshold. (B) Overlapping cliques begin forming chains via shared (k1)-node overlaps. (C) A giant percolation cluster spans much of the network when the threshold is exceeded. (D) A phase transition in $\phi(k)$, the fraction of nodes in the percolation cluster emerges as edge density increases, indicating the critical threshold $p_c(k)$ for percolation.
